# The Institutional Worker

**Published:** 1912-11-09

**Authors:** 


THE
### INSTITUTIONAL WORKER
Being a Special Supplement to "The Hospital"

### Editor's Notices.

**Contributions:**

Contributions should be written, or preferably typed, on one side of the paper only, and all articles sent in are accepted upon the distinct understanding that they are forwarded to The Hospital only.

The Editor cannot undertake to return MSS. not used, but when a stamped directed envelope is enclosed the MSS. may be returned if a special request is made.

Accepted articles and paragraphs of news will be paid for after publication at the scale rate.

**Address:**

To prevent delay all contributions and letters on editorial business must be addressed exclusively to the Editor, "The Hospital," 29 Southampton Street, Strand, London, W.C. It is important that this regulation shall be strictly observed.

**Correspondence:**

Correspondence on all subjects is invited. The name and address of correspondents must be given as a guarantee of good faith, but not necessarily for publication.

**Special Articles:**

Special articles are invited, and questions, inquiries, and paragraphs upon all matters relating to the work, administration, and management of general, special, mental, fever, cottage and convalescent hospitals, sanatoria, homes, institutions, societies and organisations for the treatment or care of the sick, injured and dependants of all classes. Every matter affecting the interests and welfare of the staff of all grades working in these institutions will receive special consideration.

**Administrative Medicine, Research and Items of News:**

Special terms will be paid for approved articles on subjects in this wide field by experts who have made some section of it their special study and interest. We wish to give prominence to every movement tending to advance accuracy of diagnosis, efficiency in treatment, and the development of modern methods to eradicate disease and promote the welfare of the suffering.

**A Bureau of Information:**

We invite inquiries and applications for information and help in providing for that numerous class of sufferers who require special care or house-room which they cannot obtain in their own homes or secure for themselves. **We cannot, however, prescribe, or recommend practitioners.**

**Books for Review:**

Publishers are particularly requested to send advance proofs of any new books of importance whenever possible, as the Editor has made arrangements to publish immediate reviews on a new plan.

**Photographs, Plans, Blocks, and Illustrations:**

It is requested that wherever possible MSS. may be accompanied by illustrations in any of the above forms. The name of the sender and of the article to which it belongs should be written on the back of each photograph, drawing, or block for purposes of identification.

**Local Papers:**

Newspapers containing reports or news paragraphs should be marked and addressed to the Sub-Editor.

**The Coupon System:**

A coupon will be found at the bottom of the third inside page of the cover each week. This coupon must be attached to every question or inquiry to which an answer is desired in The Hospital.

### Patients and Approved Homes.

All who are sick or in difficulties have the opportunity of making their wants known free of charge under a pseudonym in The Hospital Bureau of Information, and can thus be brought under the notice of those willing and able to render assistance. A large number of the Nursing Homes on the Approved List have generously signified their willingness to receive patients in poor circumstances recommended to them through the Bureau at greatly reduced terms. No charge is made on either side for the introduction of patients, and the advantages afforded by this free intercommunication between patients and Homes of tried efficiency should be noted by members of the medical and nursing professions. A descriptive booklet of The Hospital Information Bureau will be forwarded free on application to the Editor,

The Hospital Bureau of Information,
28 and 29 Southampton Street,
Strand, London, W.C.

### Manager's Notices.

**All letters on business matters must be addressed to The Manager, "The Hospital," 28 and 29 Southampton Street, London, W.C.**

**Subscriptions,** which may commence at any time, are payable in advance. Cheques and Post-Office Orders should be crossed London County and Westminster Bank, Covent Garden Branch, and made payable to the Manager of The Hospital.

Rates of Subscription (including Postage).

| *United Kingdom.* | | *Foreign and Colonial.* | |
|---|---|---|---|
| Three Months ... | ... 2s. 0d. | Three Months ... | ... 2s. 6d. |
| Six Months ... | ... 4s. 0d. | Six Months ... | ... 5s. 0d. |
| Twelve Months ... | ... 6s. 6d. | Twelve Months ... | ... 8s. 8d. |

*SUBSCRIPTION FORM.*

*Please send me* The Hospital *for*..................*months, commencing with your issue of*............................., *for which please find enclosed*.............................

*Name*..................................................

*Address* .........................................................

.......................................

*Date*....................................

*To* ................................... *Newsagent.*

**Advertisements:**

To ensure insertion, all advertisements for The Hospital must reach the Manager not later than Wednesday at noon in each week. For scale of charges see pages vii and 2.

**Remittances:**

**It is especially requested that remittances be made by Cheque or Postal Order, and in halfpenny stamps only when the amount is under sixpence.**

THE MEDICAL LIBRARY.

Post Free.

**Photomicrography.** A Practical Guide and Introduction. 41 Reproductions of original specimens. 63 Drawings illustrating instruments and methods. By EDMUND SPITTA, M.R.C.S., F.R.A.S. ... ... ... ... 12s. 0d.

**Clinical Diagnosis.** A Practical Illustrated Handbook of Chemical and Microscopic Methods. By W. G. AITCHISON ROBERTSON, M.D., F.R.C.P. ... ... ... ... 3s. 9d.

**Medical History from the Earliest Times.** By E. WITHINGTON, M.A., M.B. Oxon ... ... 12s. 10d.

**Notes on General Practice.** Danger Signs and Common Pitfalls. By S. M. HEBBLETHWAITE, M.D. ... ... ... ... ... 3s. 9d.

*Of all Booksellers, or of*

THE SCIENTIFIC PRESS, LIMITED,
28 & 29 Southampton Street, Strand, London, WC

Books for Institutional Workers.

"Midwife's Pocket Dictionary, with revised Rules of the C.M.B." ... ... ... ... ... ... 1s. 1d.
"The Nurses' Pronouncing Dictionary" ... ... 2s. 0d.
"A Handbook for Nurses." (J. K. Watson, M.D.) 5s. 4d
"Art of Feeding the Invalid." ... ... ... ... 1s. 6d.
"Surgical Ward Work and Nursing." ... ... ... 5s. 5d
"Massage Movements, including the Nauheim Exercises" ... ... ... ... ... ... ... 1s. 1d.
"Surgical Instruments and Appliances used in Operations" (Harold Burrows, M.B.London, B.S., F.R.C.S.) ... ... ... ... ... 1s. 8d.
"The Nurse's Materia Medica" (Herbert French, M.D.) ... ... ... ... ... ... 2s. 9d.

Case-papers, Charts, Professional Cards, and every kind of Institutional Literature.

Of all Booksellers or of The Scientific Press, Limited, 28 and 29 Southampton Street, Strand, London, W.C.

A LIST of the NEW PUBLICATIONS of the SCIENTIFIC PRESS, LIMITED, will be sent post free to any member of the Medical Profession on receipt of post card.

28 & 29 SOUTHAMPTON STREET, STRAND, LONDON, W.C.

